# Adverse Effects of Sarcopenic Obesity on Postoperative Complications after Major Hepatectomy in Patients with Hilar Cholangiocarcinoma

**DOI:** 10.3390/jcm11071860

**Published:** 2022-03-27

**Authors:** Okjoo Lee, Yong Chan Shin, Youngju Ryu, Sang Hyun Shin, Jin Seok Heo, Chang-Sup Lim, In Woong Han

**Affiliations:** 1 Samsung Medical Center, Division of Hepatobiliary-Pancreatic Surgery, Department of Surgery, Sungkyunkwan University School of Medicine, 81, Irwon-Ro, Gangnam-gu, Seoul 06355, Korea; altanis7@gmail.com (O.L.); honeymoneykr@naver.com (Y.R.); surgeonssh@gmail.com (S.H.S.); jsheo.md@gmail.com (J.S.H.); 2 Department of Surgery, Ilsan Paik Hospital, Inje University College of Medicine, Goyang 10380, Korea; ycshindr@gmail.com; 3 Department of Surgery, Seoul Metropolitan Government-Seoul National University Boramae Medical Center, Seoul National University College of Medicine, 20, Boramae-ro 5-gil, Dongjak-gu, Seoul 03088, Korea

**Keywords:** sarcopenic obesity, hilar cholangiocarcinoma, postoperative complication

## Abstract

Sarcopenic obesity (SO), which is defined as a high ratio of visceral adipose tissue to skeletal muscle, is a well-known risk factor for post-hepatectomy outcomes in patients with hepatocellular carcinoma. However, few studies have evaluated the effect of SO on postoperative outcomes in patients with hilar cholangiocarcinoma (CCC). This retrospective study aimed to evaluate the effect of preoperative SO on postoperative outcomes in patients with hilar CCC following major hepatectomy. Preoperative SO was assessed in 328 patients undergoing hepatectomy for hilar CCC at three institutions between 2006 and 2016. SO was calculated from cross-sectional visceral fat and muscle area displayed on preoperative CT imaging. Preoperative SO was present in 98 patients (29.9%). The major complication rate in patients with SO was higher than in those without SO (54.1% vs. 37.0%, *p =* 0.004). Additionally, postoperative hospital stays were prolonged in patients with SO (18.5 vs. 16.5 days, *p =* 0.038). After multivariable analysis, SO was identified as an independent risk factor for major complications after hepatectomy in hilar CCC patients (OR = 0.866, 95% CI: 1.148–3.034, *p =* 0.012). Careful postoperative management is needed after major hepatectomy in hilar CCC patients with SO.

## 1. Introduction

In recent years, hepatectomy has been gradually and more frequently performed on elderly patients with many comorbidities, prompting the need to exactly define patients who are able to undergo major hepatectomy and may overcome potential severe morbidities. There are many factors that affect the outcomes of major hepatectomy, and sarcopenia is one of the most common risk factors for physical disability, poor quality of life, and patient survival [1,2]. Sarcopenia is characterized as the progressive or generalized loss of skeletal muscle mass and strength with or without increased fat mass [3]. However, because of asymptomatic features, the ideal time for the proper management of sarcopenia can be missed. 

For this reason, preoperative evaluation of body composition parameters via computed tomography (CT) scan images probably improves risk management [4]. As shown in a meta-analysis, radiologically determined sarcopenia patients who underwent major abdominal surgery had a poor prognosis for advanced postoperative complications and patient survival [5]. Moreover, sarcopenia patients not only had longer ICU and hospital admission stay lengths, but they also had greater hospitalization costs [6].

Presently, there is a concurrence of both obesity and sarcopenia, a condition known as sarcopenic obesity (SO), which is characterized by a high visceral adipose tissue to skeletal muscle ratio [7]. Reduced lean mass with an excess percentage of body fat was one of the first SO definitions. Whether the effects of sarcopenia and obesity are cumulative or whether an interaction exists is unclear. However, SO is related to metabolic problems in general and is associated with a higher risk of respiratory disease incidence and greater cardiovascular mortality [8,9]. Moreover, as shown in some studies, SO is a risk factor for higher morbidity, mortality, and length of stay in major abdominal elective and emergency surgery cases [10,11,12].

Recently, SO has attracted attention for its potential to be a prognostic factor for patients with various types of hepato-biliary-pancreatic cancers such as gallbladder cancer, pancreas cancer, and hepatocellular carcinoma [13,14,15]. However, there have been few reports on the relationship between SO and postoperative outcomes in patients with hilar cholangiocarcinoma (CCC). Therefore, this study aimed to evaluate the effect of preoperative SO on postoperative outcomes in patients with hilar CCC following major hepatectomy.

## 2. Methods

### 2.1. Patients and Data Collection

From 2006, all data from patients with hilar CCC who underwent surgery at Samsung Medical Center, Seoul Metropolitan Government-Seoul National University Boramae Medical Center, and Ilsan Paik Hospital were prospectively collected in an electronic database. Among these, data from patients who underwent curative-intent major hepatectomy (including hemihepatectomy or trisectionectomy with caudate segmentectomy) for hilar CCC between January 2006 and December 2016 were extracted and reviewed retrospectively after IRB approval (number: 2018-06-139). Patients operated on for metastatic cancer or double primary cancer were excluded from this study. During this period, 357 patients were treated for perihilar CCC with major hepatectomy at one of the above hospitals. Of these patients, 328 patients were eligible for computed tomography (CT) scan evaluation for diagnosis of sarcopenia. All data were reviewed for postoperative complications and overall survival (OS) as well as for clinical and pathological data.

### 2.2. Preoperative CT Scan Evaluation

All CT images were evaluated at the portal phase of the CT scan. The program used in this study was developed in the Department of Radiology at Samsung Medical Center and allowed for evaluation of muscle, visceral fat, and subcutaneous fat, and CT images of body composition were evaluated using MATLAB version R2010a (MathWorks Inc., Natick, MA, USA) [16]. Muscle, visceral fat, and subcutaneous fat were identified on pre-processed CT images using fuzzy c-means clustering algorithms. The pre-processed CT images were then segmented into inner and outer CT images using the identified muscle boundary. Pixels in the outer CT images were segmented into four clusters based on the Hounsfield unit (HU) of pixels using the fuzzy c-means clustering algorithm. These clusters included pixels in the muscle, subcutaneous fat, bone, and background. Pixels in the visceral fat were confirmed using HU thresholds of −300 to −50 in the inner CT images. The areas were evaluated in the L3 level by multiplying the number of pixels by the pixel surface area for each type (i.e., muscle, visceral fat, and subcutaneous fat).

### 2.3. Definitions of Sarcopenia and Sarcopenic Obesity

Moon et al.’s definition of sarcopenia was evaluated using the skeletal muscle index (SMI = skeletal muscle area at L3/height^2^: <50.18 cm^2^/m^2^ for men and SMI < 38.63 cm^2^/m^2^) for women [17]. These are standard values determined in a large cohort study conducted by a Korean national institution and were appropriate for use in this study [17]. Visceral obesity was defined as visceral fat area (VFA), whereas SO was defined by VFA/SMI. Cut-off values for visceral obesity and SO were evaluated using the receiver operating characteristics (ROC) curve. According to the ROC curve, the cut-off value of visceral obesity was 113.8 cm^3^, and the cut-off value of SO was 2.5 m^2^. Both sensitivity and specificity values exceeded 60% and had statistical significance (data not shown).

### 2.4. Surgical Technique and Postoperative and Oncologic Outcomes

All major hepatectomies were performed by experienced attending surgeons in three centers. For patients with moderate to severe jaundice, biliary drainage was performed before operation, and resection type was decided by extent of tumor and the relative location. If the tumor was mainly located in right or left perihilar bile ducts, tumor involvement in the liver parenchyma or unilateral hepatic artery, or portal vein was observed on preoperative images, an extended right or left hemihepatectomy or trisectionectomy including caudate lobectomy combined with bile duct resection was performed. A regional lymphadenectomy was performed on the right side of the celiac artery along with removal of all tissue in the hepatoduodenal ligament except for the portal vein and the hepatic artery. Postoperative outcomes were evaluated during hospital admission and within 30 days after discharge. Postoperative complications were defined and classified according to the Clavien–Dindo classification (CDC) [18]. CDC grade ≥ III was defined as a major complication. Post-hepatectomy liver failure (PHLF) was defined according to the International Study Group of Liver Surgery (ISGLS) [19].

### 2.5. Statistical Analyses

Between-group differences of mean values with interquartile range were compared with independent *t*-tests, and between-group differences of numbers and percentages were compared with χ^2^ test or Fisher’s exact test. Univariable and multivariable logistic regression model was performed to determine the relationships between variables and surgical outcomes. Variables with a *p*-value < 0.10 in univariate analyses were included in the multivariate analysis. Kaplan–Meier survival curves were used to calculate OS. Cox regression analysis was used to confirm predictive values for OS. A *p*-value < 0.05 was considered statistically significant. All statistical analyses were performed using SPSS version 23.0 (IBM, Armonk, NY, USA).

## 3. Results

### 3.1. Clinical Characteristics of Sarcopenic Obesity

The characteristics of patients in the SO group (*n =* 98, 29.9%) are shown in Table 1. The proportion of patients with SO was significantly higher among men (34.1% vs. 21.3%, *p =* 0.017). Patients with SO had a higher body mass index (BMI) compared with patients without SO (24.6 vs. 22.8, *p <* 0.001). There were no significant differences in laboratory and operative findings between the SO and non-SO groups.

### 3.2. Postoperative Outcomes in Sarcopenic Obesity

Postoperative outcomes between patients with and without SO were compared according to demographics and perioperative characteristics (Table 2). Incidences of major complications with a CDC grade ≥ III were higher in the SO group than in the non-SO group (54.1% vs. 37.0%, *p =* 0.004). Patients with SO showed higher PHLF incidence than non-SO patients, but the difference was not statistically significant (44.9% vs. 34.3%, *p =* 0.071). There were no differences between the SO and non-SO groups in other postoperative complications involving surgical site infection, intra-abdominal abscess, postoperative hemorrhage, biliary fistula, postoperative hospitalization days, or 30-day mortality (Table 2). Moreover, the five-year survival rate of the SO group was not different from that of the non-SO group (45.5% vs. 42.9%, *p =* 0.498).

Meanwhile, there were no significant differences in postoperative outcomes between the sarcopenia and non-sarcopenia groups in terms of major complications (47.4% vs. 39.8%, *p =* 0.203) involving postoperative hemorrhage, PHLF, total hospital stay length (17.6 vs. 16.9 days, *p =* 0.130), or postoperative mortality (1.0 vs. 2.2%, *p =* 0.674).

### 3.3. Risk Factor Analysis for Major Complications

Risk factor analysis of major complications with a CDC grade ≥III was performed using regression models (Table 3). In univariable analyses, male sex and SO were significantly associated with major complication occurrence. These two factors were identified as independent risk factors for major complications in multivariable analysis (male: OR = 1.937, 95% CI: 1.182–3.174, *p =* 0.009; SO: OR=1.866, 95% CI: 1.148–3.304, *p =* 0.012). However, sarcopenia was not statistically related to major complication occurrence (Table 3).

We also performed a risk factor analysis of grade B or C PHLF development (Table 4). Univariable analyses showed statistical significance in preoperative PVE, right-side hepatectomy, longer operative time, and combined portal vein resection with PHLF occurrence. After multivariable analysis, right-side hepatectomy (OR = 3.676, 95% CI: 1.833–7.371, *p <* 0.001) and longer operative time (OR = 1.004, 95% CI: 1.001–1.007, *p =* 0.008) were independent prognostic factors of PHLF (Table 4).

## 4. Discussion

Sarcopenic obesity (SO) is an important factor in hepatobiliary cancer prognosis [13,14,15]. Factors that are conventionally used to estimate cancer prognosis after surgery depend on achieving complete oncological resection (R0 resection) or on histologic examination. However, as more studies have evaluated SO as a factor influencing survival rate or complication risk, consideration of this factor is gradually being used in clinical practice. SO can be diagnosed from the preoperative CT scans that are routinely performed for CCC staging, thus causing no additional burden for patients or hospitals [20]. The Hounsfield unit average calculation (HUAC) of the muscles from preoperative CT is a measure of muscle density and fatty infiltration. Therefore, the application of HUAC has the advantage of measuring both muscle mass and quality [21].

The exact biological mechanism in SO influencing postoperative complications has not been elucidated. It is suspected that SO patients have frailty and tend to be more insulin resistant, which is suspected of promoting metabolic distress. Moreover, chronic inflammation induced by pro-inflammatory adipokines can deplete the physiological reserve needed to overcome the major complications of SO [22,23]. Therefore, it is possible that such an inflammatory response has a negative effect on the recovery period after a major hepatectomy in hilar CCC patients.

This multicenter study found that SO was associated with major complications in hilar CCC patients. Although hilar CCC incidence is small, we tried to overcome small sample sizes through a multicenter study. Consequently, our study found that preoperative SO was an independent risk factor for major complications after major hepatectomy in patients with hilar CCC (OR = 1.866, 95% CI: 1.148–3.034, *p =* 0.012), and SO patients had longer hospital stays than non-SO patients (18.5 vs. 16.5, *p =* 0.038). These findings are significant in terms of preoperative risk evaluation and management optimization for CCC patients undergoing major hepatectomy. Along with the loss of skeletal muscle, visceral obesity produces insulin resistance, which is a major risk factor for metabolic syndrome and has also been associated with major complications [22]. The correlation between fat compartments and muscle is well established and has relevant effects on metabolism; skeletal muscle probably secretes specific cytokines that play a protective role against the harmful effects of pro-inflammatory adipokines expressed in obesity [23].

Sarcopenia can be considered a prognostic factor for patients who underwent major hepatectomy for hilar CCC. Meanwhile, sarcopenia in patients with malignancy has been reported over the last decade. Recent studies have reported that sarcopenia has been identified as a poor prognostic factor for patients after several kinds of operations, including colectomy, hepatectomy, and pancreaticoduodenectomy [24,25,26]. However, in our study, there was no significant difference in complications between patients with and without sarcopenia (*p =* 0.203). Sarcopenia was not a significant risk factor for major complications or grade B or C post-hepatectomy liver failure. Few studies have been conducted that explore the relationship between sarcopenia and postoperative outcomes in CCC patients. Okumura et al. have reported that sarcopenia is a poor prognostic factor for patient survival and recurrence of malignancy in patients with extrahepatic biliary malignancies, including hilar CCC [27]. However, because their research included only a small size of patients with hilar CCC and different types of malignancies with different histological properties, it is inappropriate to suggest that the study exactly assessed the impact of sarcopenia, and larger-scale studies are needed on the postoperative outcome of patients with hilar CCC.

SO seems to have a greater effect than sarcopenia on surgical outcomes in hilar CCC patients with major hepatectomy. Otsuji et al. have reported that sarcopenia is a poor prognostic factor for morbidity in liver failure patients who underwent major hepatectomy [15]. However, few studies have explored the relationship between SO and postoperative prognosis in major hepatectomy in hilar CCC patients. Although sarcopenia has a broader definition than SO, when considered alone within a multivariable regression model, this variable did not emerge as a significant factor associated with major complications. Accordingly, if we could accurately measure pro-inflammatory adipokines in blood, we could test the above hypothesis more quantitatively. There is potential clinical evidence supporting the application of a preoperative treatment strategy comprising preoperative physical management therapy to decrease major postoperative complications in high-risk patients undergoing major hepatectomy [28]. Additionally, body composition parameters should be routinely assessed, and individually selected pathways should be implemented, including prehabilitation programs for high-risk SO patients. SO patients with poor preoperative physical status who are at high risk for major complications may be advantaged by prehabilitation programs that focus on chronic morbidity management, nutrition, and physical activity status [29,30]. Hence, considering that chemotherapy is recommended, high-risk SO patients with curable disease may even benefit from being shifted from upfront surgery to a selected preoperative evaluation that includes neoadjuvant chemotherapy and a rehabilitation strategy [31]. However, this is a study of acute complications, so we cannot draw conclusions about the effect of rehabilitation treatments. Therefore, careful preoperative and postoperative management and more observational studies are needed on patients with hilar CCC and SO after major hepatectomy. 

As mentioned above, although we found that SO was significantly associated with complications, SO did not have a significant effect on PHLF and survival. This is probably because SO is a variable that reflects overall nutritional and immune status, whereas PHLF is more affected by residual liver function and volume. Therefore, it is presumed that SO is not associated with PHLF after major hepatectomy. However, we found no significant association between SO and PHLF, which suggests that this did not affect survival. A long-term study of the influence of SO on oncological effects after major liver resection for hilar CCC would be clinically beneficial. In addition, right hepatectomy is a significant risk factor for PHLF, although hilar CCC is a disease that requires extended surgery, considering complex surgical techniques such as central hepatectomy can help oncological resections with a lower risk of PHLF.

In the postoperative outcomes, the incidence of postoperative liver failure was extremely high—over 30%. Our center does not have a special protocol for presurgical conditioning methods prior to major hepatectomy in hilar CCC patients. However, mortality within the postoperative 30 days was relatively low, and most patients were discharged without severe complications. The reason for the high PHLF is most likely transient intensive care unit treatment or the administration of albumin, which is ISGLS grade B. Of course, the patient’s condition or laboratory findings may not reach the level of clinically serious postoperative liver failure; however, the possibility that it occurred due to decreased liver function after major hepatectomy could not be excluded.

This study had some limitations. First, it was a retrospective analysis of a cohort in South Korea. Therefore, the results might not be generalizable to other countries and continents, and despite its multicenter nature, this study was performed in three high-volume academic hospitals in a similar region; therefore, outcomes may only be generalizable to the same contexts. Second, in defining sarcopenia and SO, functional aspects such as grip-strength measurement, etc., were not sufficiently estimated due to the retrospective study design. Third, the automated segmentation function made in MATLAB version R2010a was used to calculate skeletal muscle area and then manually corrected for errors, but the possibility of measurement errors cannot be entirely ruled out. Finally, patients with SO may have non-alcoholic fatty liver disease (NAFLD), which increases the risk of liver failure after major hepatectomy. Although patients diagnosed with fatty liver before major hepatectomy were rare, the analysis considering the risk of NAFLD for PHLF may have been overlooked.

In conclusion, SO was an independent risk factor for severe complications following major hepatectomy in patients with hilar CCC. Hence, SO is a better predictive factor than sarcopenia for patient prognosis. Our findings indicate that preoperative SO management for major hepatectomy can be a feasible and safe option for hilar CCC in SO patients. A large-scale, prospective, randomized study is needed to further investigate the long-term impact of SO. With the careful and definitive evaluation of SO, we expect increased patient survival and improved patient prognosis.

## Figures and Tables

**Table 1:** Patient clinical and pathological data.

Variables	Sarcopenic Obesity (*n =* 98)	Non-Sarcopenic Obesity (*n =* 230)	*p*
Age (years)	66.2 (46–82)	64.5 (42–89)	0.089
Sex			0.017
Male, *n* (%)	75 (76.5)	145 (63.0)	
Female, *n* (%)	23 (23.5)	85 (37.0)	
Body mass index, kg/m^2^	24.6 (17.4–33.5)	22.8 (14.8–40.5)	<0.001
Underlying disease			
Cardiac disease, *n* (%)	53 (56.4)	83 (36.9)	0.001
Diabetes mellitus, *n* (%)	31 (31.6)	45 (19.6)	0.018
Liver disease, *n* (%)	5 (5.1)	8 (3.5)	0.540
Charlson Comorbidity Index, *n* (%)			0.524
0–6	91 (92.8%)	219 (95.2%)	
Above 6	7 (7.2%)	11 (5.8%)	
ASA score, *n* (%)			0.709
1	15 (17.2)	41 (21.4)	
2	65 (74.7)	139 (72.4)	
3	6 (6.9)	12 (6.3)	
4	1 (1.1)	0 (0)	
CA 19–9, mg/dl	477.3 (0.10–12725.36)	417.2 (0.4–15453.39)	0.482
Neoadjuvant treatment, *n* (%)	0 (0)	5 (2.3)	0.327
Preoperative PVE, *n* (%)	13 (13.3)	40 (17.4)	0.353
Bismuth type, *n* (%)			0.115
IIIa	25 (25.5)	79 (34.3)	
IIIb	73 (74.5)	151 (65.7)	
Portal vein resection, *n* (%)	9 (9.2)	32 (13.9)	0.236
Operation time (min)	420.8 (194–1400)	394.7 (180–965)	0.054
Poorly differentiated tumor, *n* (%)	24 (24.5)	58 (25.2)	0.889
T stage, *n* (%)			0.298
T1	16 (16.3)	19 (8.3)	
T2	69 (70.4)	176 (76.5)	
T3	9 (9.2)	24 (10.4)	
T4	3 (3.1)	7 (3.0)	
N stage, *n* (%)			0.038
N0	75 (76.5)	147 (63.9)	
N1	17 (17.3)	61 (26.5)	
N2	6 (6.1)	22 (9.6)	
R1 resection, *n* (%)	8 (8.2)	32 (13.9)	0.145

Abbreviations: PVE, portal vein embolization.

**Table 2:** Postoperative complications between the sarcopenic obesity and non-sarcopenic obesity groups.

Variables	Sarcopenic Obesity (*n =* 98)	Non-Sarcopenic Obesity (*n =* 230)	*p*
Major complication (CDC grade ≥3), *n* (%)	53 (54.1)	85 (37.0)	0.004
Surgical site infection, *n* (%)	27 (27.6)	60 (26.1)	0.786
Intraabdominal abscess, *n* (%)	16 (16.3)	32 (13.9)	0.571
Postoperative hemorrhage, *n* (%)	3 (6.0)	9 (7.3)	0.999
Biliary fistula, *n* (%)	6 (6.1)	16 (7.0)	0.782
Post-hepatectomy liver failure ^a^, *n* (%)	44 (44.9)	79 (34.3)	0.071
Postoperative hospital stay, days	18.5 (4–88)	16.5 (7–82)	0.038
Postoperative mortality (<30days)	3 (3.1)	3 (1.3)	0.369

^a^ ISGLS grade B or C.

**Table 3:** Risk factor analysis for major complications.

Variables	Univariable Analysis			Multivariable Analysis		
	Odds Ratio	95% CI	*p*	Odds Ratio	95% CI	*p*
Age	0.983	0.957–1.009	0.190			
Male (vs. female)	2.075	1.274–3.379	0.003	1.937	1.182–3.174	0.009
Body mass index	1.030	0.960–1.106	0.409			
Diabetes mellitus	1.103	0.704–1.729	0.668			
Combined liver diseases	1.324	0.790–2.217	0.286			
ASA score (≥3)	2.277	0.728–7.117	0.147			
Preoperative PVE	1.816	0.694–4.753	0.219			
Bismuth type IIIa	1.067	0.589–1.931	0.831			
Bismuth type IIIb	1.622	0.878–2.998	0.122			
Operative time	1.330	0.806–2.193	0.264			
Preoperative sarcopenia	1.001	0.999–1.003	0.293			
Preoperative Sarcopenic obesity	2.009	1.244–3.244	0.004	1.866	1.148–3.034	0.012

Abbreviations: CI, confidence interval; PVE, portal vein embolization.

**Table 4:** Risk factor analysis for grade B or C post-hepatectomy liver failure.

Variables	Univariable Analysis			Multivariable Analysis		
	Odds Ratio	95% CI	*p*	Odds Ratio	95% CI	*p*
Age	1.015	0.988–1.042	0.284			
Male (vs. female)	1.573	0.964–2.568	0.069	1.385	0.783–2.449	0.263
Body mass index	0.992	0.923–1.067	0.831			
Cardiac disease	0.991	0.627–1.567	0.970			
Diabetes mellitus	1.198	0.709–2.024	0.499			
Combined liver diseases	0.732	0.221–2.429	0.773			
ASA score (≥3)	1.002	0.376–2.669	0.997			
Preoperative PVE	3.081	1.682–5.642	<0.001	1.836	0.925–3.643	0.082
Bismuth type IIIa	4.067	2.208–7.490	<0.001	3.676	1.833–7.371	<0.001
Bismuth type IIIb	1.549	0.812-2.953	0.184			
Operative time	1.003	1.001–1.006	0.011	1.004	1.001–1.007	0.008
Combined portal vein resection	2.132	1.012–4.125	0.022	1.529	0.709–3.301	0.279
Preoperative Sarcopenia	1.040	0.638–1.695	0.876			
Preoperative Sarcopenic obesity	1.557	0.962–2.522	0.071	1.396	0.801–2.433	0.239

Abbreviations: CI, confidence interval; PVE, portal vein embolization.

## Data Availability

The data that support the findings of this study are available from the corresponding author upon reasonable request.
